# Inadvertent Intravenous Administration of Milk in Postoperative Intensive Care Unit: Sequelae and Management

**DOI:** 10.7759/cureus.16154

**Published:** 2021-07-04

**Authors:** Rajesh Panda, Pankaj Kundra, Pooja Singh, Divya Hirolli, Priyadarshani Padhihari

**Affiliations:** 1 Department of Anesthesiology and Critical Care, All India Institute of Medical Sciences, Bhopal, IND; 2 Department of Anesthesiology and Critical Care, Jawaharlal Institute of Postgraduate Medical Education and Research, Puducherry, IND; 3 Department of Anesthesiology and Critical Care, All India Institute of Medical Sciences, New Delhi, IND; 4 Department of Anesthesiology, Apollo Hospitals, Bhubaneswar, IND

**Keywords:** feeding jejunostomy, tubing misconnection, intravenous, ventilation-perfusion scan, intensive care

## Abstract

Tubing misconnections is an unfortunate and rare occurrence in intensive care units, but the complication is grossly underreported as it is often attributed to human error rather than device failure. This potential underreporting of a complication causes concern because it can be prevented by making an appropriate device design and increase awareness among health care workers. In this case report, we have discussed an enteral feed misconnection to an intravenous cannula has led to respiratory distress and acute kidney injury in a patient admitted to the postoperative intensive care unit. We propose a standard operating protocol for management in such a scenario and the role of ventilation-perfusion (V/Q) scan as an alternative to conventional computed tomography pulmonary angiogram (CTPA) in acute kidney injury patients.

## Introduction

The definition of medical misconnections includes seemingly obvious incompatible systems that, when inadvertently connected, can result in life-threatening events in the clinical arena [1]. Enteral nutrition (EN) is defined as nutrition provided through the gastrointestinal (GI) tract via a tube, catheter, or stoma to deliver nutrients distal to the oral cavity [2]. This case report focuses on enteral misconnections with intravenous port. In such cases, serious patient harm, including death, can occur if fluids, medications, or nutritional formulas intended for administration into the GI tract are administered intravascularly [3]. As with other voluntary adverse event reporting systems, the reporting of enteral misconnections may greatly underrepresent the number of actual cases.

## Case presentation

A 23-year-old male with multiple stab wounds to the abdomen presented to a tertiary care hospital in Southern India. Radiological imaging revealed perforation of the duodenum (D1-D2 junction) with a right hemothorax. A right-sided intercostal drain was placed in the emergency medical services, after which the patient was transported to the operating theatre for exploratory laparotomy. Surgical primary repair of the perforation with omental patch closure was accomplished, and a feeding jejunostomy was placed under general anesthesia uneventfully. The patient was transferred to the surgical intensive care unit for elective ventilation.

Tracheal extubation was accomplished on post-operative day 1, and the patient was recovering well. On the fourth postoperative day, he developed sudden breathlessness, tachypnea (respiratory rate 32 breaths/min), tachycardia (heart rate 110 beats/min) and hypotension (80/60 mmHg), as measured via non-invasive blood pressure, following administration of enteral feeds (milk). On examination, it was noticed that the enteral feed port was inadvertently connected to the peripheral intravenous catheter instead of feeding jejunostomy, and 20 minutes had elapsed when the problem was identified. The enteral feed was immediately stopped, but approximately 30 mL had already been administered. The patient was transferred to a multidisciplinary critical care unit (CCU). Oxygen therapy was initiated through a face mask (5 L/min), and hypotension was managed by fluid resuscitation with 500 mL bolus of plasmalyte and continued at 100 mL/h. Despite fluid resuscitation, the systolic blood pressure dropped to 70 mmHg, for which Inj. Noradrenaline infusion (0.1 μg/kg/min) was started, and Inj. Hydrocortisone 100 mg was administered intravenously. Direct arterial pressure monitoring was established after right radial artery catheterization; subsequently, noradrenaline infusion was titrated to maintain a mean arterial pressure (MAP) of 75 mmHg. Arterial blood gas analysis showed pH 7.28, pCO_2_ 28 mmHg, pO_2_ 62 mmHg, HCO_3_ 20 meq/L, lactates 1.2 mmol/L and hematocrit 32. The patient continued to be tachypneic (respiratory rate > 35-40 breaths/min), and his oxygen saturation fell to 90%. In view of the deteriorating status, invasive ventilation was chosen. Rapid sequence tracheal intubation was performed using intravenous ketamine (30 mg) and propofol (20 mg) with apneic oxygenation of 15 L/min via nasal cannula during laryngoscopy. Tracheal intubation was achieved with injection suxamethonium 2 mg/kg. Ventilatory support was initiated using synchronized intermittent mandatory ventilation (SIMV) with pressure support (PS) mode. The ventilatory parameters were set to deliver ventilatory breaths at a pressure of 18 cm H_2_O, at a respiratory rate of 16 breaths/min. A PS of 12 cm H_2_O was used to support intervening spontaneous breaths. Peak end-expiratory pressure (PEEP) was set at 6 cm H_2_O with fractional inspired concentration (FiO_2_) of 0.5. Sedation was initiated with dexmedetomidine infusion at 30 μg/h after administering a bolus dose of 25 μg. Prophylactic broad-spectrum intravenous antibiotics in the form of Inj. Cefoperazone with sulbactam (2 gm twice daily), Inj. Clindamycin (600 mg twice daily) and Inj. metronidazole 500 mg six hourly) were started after taking a sample for blood culture. Additionally, prophylactic thromboprophylaxis was initiated with Inj. Enoxaparin 40 mg once daily. Laboratory parameters including liver function test and renal function test were within normal range. Bedside 2D trans-thoracic echocardiography was done, which showed normal chamber size and good contractility. Furthermore, to rule out pulmonary embolism, we opted for a Tc-99m macro aggregated albumin (MAA) lung perfusion study over CTPA to prevent renal insult as creatinine values had increased by greater than 0.3 mg/dL in the last 48 h (Video 1). Ventilation-perfusion study showed a match perfusion defect in the lower lobe of the right lung the lower right-lung lobe secondary to hemothorax, with no evidence of pulmonary embolism. The hemodynamic parameters gradually improved, and the patient was weaned off noradrenaline gradually over two days. After the sedation was stopped, the patient became fully conscious with Glasgow coma scale (GCS) of E4VTM6. Extubation was accomplished on the third day of CCU stay after passing a spontaneous breathing trial for one hour on T-piece. Inj. Hydrocortisone 50 mg was given thrice daily for four days and stopped one day after extubation. Blood cultures were sterile, and antibiotics were de-escalated after seven days. Enoxaparin prophylaxis was stopped after 10 days.

**Video 1:** Tc-99m MAA lung perfusion scan showing complete perfusion of all lung fields with no evidence of Pulmonary Embolism MAA - macro aggregated albumin

## Discussion

There are few reports of medical injury due to catheter misconnections in the literature; however, they are only the tip of the iceberg worldwide [3,4]. Enteral feed-in intravenous lines run the risk of anaphylaxis, pulmonary embolism, acute kidney injury (AKI) and bacteremia. Moreover, the high osmolarity can cause thrombosis of veins, particularly if given in a peripheral venous line, as was in this study. Thromboembolic complications, AKI and anaphylaxis may be explained by micro-embolism of fat globules and water-insoluble particles, as well as an immune response to foreign antigens [5,6]. The size of particulate matter is an important factor when considering the potential risk to patients. According to Walpot et al., particles as small as 2 μm in diameter have been associated with the microthrombi formation in patients [7]. Akers et al. noted that the smallest capillary blood vessels are considered to have a diameter of approximately 7 μm [8]. Therefore, all particles having a size ≥7 μm can conceivably become entrapped in and occlude capillaries, increasing potential adverse effects. Bacteremia may be explained by the high bacterial loads present in the milk.

We designed a standard operating protocol (SOP) for the treatment of inadvertent intravenous administration of an enteral feed. We propose the following steps be considered for effective management:

1) Immediately stop the inadvertent infusion as soon as it is recognized.

2) The patient should be shifted to the intensive care unit for continuous monitoring.

3) The patient’s relative should be informed about the complication and reassured.

4) Oxygen supplementation should be continued with ventilator backup ready in case develops respiratory failure.

5) Hemodynamic parameters should be monitored. Fluid resuscitation and inotropic support should be initiated to target MAP of 75 mmHg, in order to maintain adequate perfusion to all vital organs.

6) Serial arterial blood gas analysis will be required to monitor the adequacy of oxygenation, ventilation and metabolic status.

7) Paired blood culture samples should be collected and sent for microbiological evaluation.

8) Empirical broad-spectrum antibiotics should be started.

9) Bedside lung ultrasound and trans-thoracic echocardiography can be performed to look for right ventricular strain or dilation features to rule out pulmonary embolism.

10) If the patient is stable, definitive investigation like CTPA can rule out pulmonary embolism. If the patient has AKI, as in this study, a lung perfusion scan can be an option.

11) Prophylactic anticoagulation should be started.

In order to prevent inadvertent misconnections, several measures have been advised, such as checking for proper placement and flushing of the enteral tube before initiation of feeds. Further, standardized color coding and labeling of enteral tubes and high-risk catheters, tracing all lines from the origin to the connection port, avoiding the use of standard Luer-connection syringes and adaptors to give medication or feeds should be encouraged to avoid complications in intensive care units [9,10].

## Conclusions

Inadvertent enteral feed administration into an intravenous line can be a life-threatening iatrogenic complication. The authors recommend an SOP for the management of such avoidable complications in intensive care units and suggest a lung perfusion scan as an alternative to conventional CTPA in AKI patients. Constant vigilance, regular training and practice of safety measures to avoid inadvertent misconnections can go a long way to avoid these preventable complications.

## References

[1] (2006). Tubing misconnections--a persistent and potentially deadly occurrence. Jt Comm J Qual Patient Saf.

[2] Teitelbaum D, Guenter P, Howell WH, Kochevar ME, Roth J, Seidner DL (2005). Definition of terms, style, and conventions used in A.S.P.E.N. guidelines and standards. Nutr Clin Pract.

[3] Guenter P, Hicks RW, Simmons D (2008). Enteral feeding misconnections: a consortium position statement. Jt Comm J Qual Pat Saf.

[4] Wallace JR, Payne RW, Mack AJ (1972). Inadvertent intravenous infusion of milk. Lancet.

[5] Ryan CA, Mohammad I, Murphy B (2006). Normal neurologic and developmental outcome after an accidental intravenous infusion of expressed breast milk in a neonate. Pediatrics.

[6] Ulicny KS Jr, Korelitz JL (1989). Multiorgan failure from the inadvertent intravenous administration of enteral feeding. JPEN J Parenter Enteral Nutr.

[7] Lehr HA, Brunner J, Rangoonwala R, Kirkpatrick CJ (2002). Particulate matter contamination of intravenous antibiotics aggravates loss of functional capillary density in postischemic striated muscle. Am J Respir Crit Care Med.

[8] Akers MJ (1994). Parenteral Quality Control: Sterility, Pyrogen Particulate and Package Integrity Testing, 2nd ed. 2nd ed. Marcel Dekker, New York, NY.

[9] Cohen MR, Smetzer JL (2012). Avoiding inadvertent intravenous injection of oral liquids; medication within intravenous tubing may be overlooked; searching by drug name gives information on wrong drug. Hosp Pharm.

[10] (2007). Avoiding catheter and tubing mis-connections. Jt Comm J Qual Patient Saf.

